# Epigenetic clocks may come out of rhythm—implications for the estimation of chronological age in forensic casework

**DOI:** 10.1007/s00414-020-02375-0

**Published:** 2020-07-14

**Authors:** Barbara Elisabeth Koop, Alexandra Reckert, Julia Becker, Yang Han, Wolfgang Wagner, Stefanie Ritz-Timme

**Affiliations:** 1 Institute of Legal Medicine, University Hospital Düsseldorf, 40225 Düsseldorf, Germany; 2 Helmholtz-Institute for Biomedical Engineering, Stem Cell Biology and Cellular Engineering, RWTH Aachen Faculty of Medicine, Aachen, Germany

**Keywords:** Epigenetic clocks, Forensic age estimation, Chronological age, Confounding factors

## Abstract

There is a growing perception that DNA methylation may be influenced by exogenous and endogenous parameters. Knowledge of these factors is of great relevance for the interpretation of DNA-methylation data for the estimation of chronological age in forensic casework. We performed a literature review to identify parameters, which might be of relevance for the prediction of chronological age based on DNA methylation. The quality of age predictions might particularly be influenced by lifetime adversities (chronic stress, trauma/post-traumatic stress disorder (PTSD), violence, low socioeconomic status/education), cancer, obesity and related diseases, infectious diseases (especially HIV and *Cytomegalovirus* (CMV) infections), sex, ethnicity and exposure to toxins (alcohol, smoking, air pollution, pesticides). Such factors may alter the DNA methylation pattern and may explain the partly high deviations between epigenetic age and chronological age in single cases (despite of low mean absolute deviations) that can also be observed with “epigenetic clocks” comprising a high number of CpG sites. So far, only few publications dealing with forensic age estimation address these confounding factors. Future research should focus on the identification of further relevant confounding factors and the development of models that are “robust” against the influence of such biological factors by systematic investigations under targeted inclusion of diverse and defined cohorts.

## Introduction

In a globalized world, age estimation is an increasingly important task in Forensic Medicine. Indications for age estimation may be the clarification of unclear or unknown ages in living young migrants without valid identity documents, the identification of unknown deceased or the identification of the donor of a blood trace. Any indication for age estimation in a forensic context requires the use of the most accurate methods as well as the knowledge of error sources and influencing factors [1].

The description of age dependent DNA methylation patterns (“epigenetic clocks”) [2–5] opened up new possibilities for developing innovative methods for age estimation. Numerous groups have been working on the development and optimization of these methods, and several models for the estimation of chronological age based on DNA methylation have been established, all of which are based on different numbers and combinations of CpG sites (for review see [6]). One of the best known models was developed by Horvath [2] and includes 353 CpG sites. This model is further referred to as “Horvath clock”.

The terms “epigenetic clock”, “epigenetic age” and “epigenetic age estimation” are used somewhat differently in different scientific contexts. Their use in this review is derived from its topic “forensic age estimation”. The aim of forensic age estimation is the (as precise as possible) estimation of chronological age. Models for the estimation of chronological age based on DNA methylation (“epigenetic age estimation”) reveal age estimates (“epigenetic ages”) that may differ from the chronological age, since DNA methylation is influenced by numerous factors during the complex processes of biological ageing. At the same time, “epigenetic age” reflects biological age that may be more or less correlated with the chronological age.

The primary aim of research in the field of forensic epigenetic age estimation is to improve the accuracy of age estimation—either of living persons, deceased persons, or in the analysis of blood traces. In living persons, samples providing enough DNA in good quality can be collected easily, whereas quantity and quality of DNA in postmortem cases and traces in crime scene investigations are often restricted [7]. Quantity and quality of available DNA are crucial for the number of CpGs that can be analysed, and for the choice of the analysis technology. Prediction models developed from the analysis of high-quality and high-quantity DNA samples may not easily be transferred to case work with low-quality and low-quantity DNA from traces. Another problem in optimization of methods for epigenetic age estimation is the method-to-method-bias; model data produced with method A may not be suitable for predicting age from a case sample based on data produced with method B. Regarding this, it is important to understand that the methods used for epigenetic age predictions as well as the choice of relevant CpG sites, have major impact on the accuracy of epigenetic age estimation. We have recently demonstrated an alternative selection of 65 CpGs for epigenetic age predictions with Illumina BeadChip arrays. Furthermore, we have compared different methods for targeted analysis of age-associated DNA methylation changes with pyrosequencing, droplet digital PCR, and bisulfite barcoded amplicon sequencing [8]. Thus, we are aware that the choice of methods and clocks needs to be critically evaluated for the specific application.

Further questions arise from the biological context, since complex biological processes may have a relevant influence on DNA methylation. Apart from considering the aspects named above, the interpretation of DNA methylation data for age estimation requires—knowledge of biological confounding factors that may be of relevance in a forensic setting. This review focusses on these biological confounding factors.

Forensic research is already aware of the influence of exogenous and endogenous factors on the estimation of chronological age by epigenetic age estimation models, and a few studies have already reported a relevant impact of selected factors as ethnic factors, physical exercise and diseases [9–12] (for details see below). Spolnicka et al. [11] rightly pointed out that “…studies aiming to identify all potential players influencing differences in DNA methylation at particular loci between individuals at the same chronological age are important …for better accuracy of age prediction models. Exploration of this issue is important for age prediction reliability in routine forensic investigation”.

There are a barely manageable number of clinical and basic science publications dealing with exogenous and endogenous factors affecting DNA methylation. This review gives an overview of potential influencing factors that may actually be relevant in forensic casework and is intended to contribute to the conception of future research on the development of “specialized clocks” [13] for forensic age estimation.

We selected publications that highlight confounding factors in epigenetic age estimation and may be relevant in forensic casework; because of their relevance in forensic practise, we focussed on buccal swabs, saliva and blood as sources of DNA. Although we included only studies with reasonable sample sizes (see Tables 1 and 2) and a study design that allowed the identification of confounding factors, we are aware that some of the effects cited below have not been conclusively proven yet. Moreover, most models cited in the following text and in Tables 1 and 2 primarily focus on a better understanding of biological contexts, of biological ageing, and on the biological effects of the investigated factors—and were not developed for the estimation of chronological age. Thus, an overestimation of the effects of the named confounding factors on models developed for the estimation of chronological age in a forensic context is possible. Despite these methodological limitations, the factors listed in Tables 1 and 2 should at least be discussed as potential confounding factors as long as the risk of influence cannot be excluded.

**Table 1:** Repeatedly and consistently reported factors with effects on epigenetic age. *K* kind of sample: *b* blood, *s* saliva, *l* leukocytes, *m* monocytes, *mn* mononuclear cells, *bc* buccal cells, *bu* buffy coat

Factor	Reference	Age prediction model (referring to chronological (c), biological (b), phenotypic (p) age)	K	Information on investigated populations (as far as available)	Influence on the estimation of chronological age
Trauma/PTSD /lifetime stress	[14–20]	[14–16, 19, 20]: Horvath (353 CpGs)^c^, [16, 17, 20]: Hannum (71 CpGs)^b^	b	[14]: *n* = 517 traumatized versus *n* = 6780 non-traumatized [15]: *n* = 32 with low versus *n* = 67 with high trauma (Dutch military personnel) [16]: *n* = 281 veterans of the conflicts in Iraq and Afghanistan [17]: *n* = 339 trauma-exposed veterans [18]: review [19]: *n* = 48 PTSD versus *n* = 48 non-PTSD Vietnamese [20]: metastudy of several cohorts; *n* = 855 lifetime PTSD versus *n* = 516 lifetime controls, *n* = 876 current PTSD versus *n* = 1228 controls	Inconsistent; **↑** (*trend;* ~ 2 years)
Low socioeconomic status/education	[21–33]	[21, 23–28, 31–33]: Horvath (353 CpGs) ^c^, [21–23, 25, 27, 28, 30–33]: Hannum (71 CpGs)^c^, [27, 29]: Levine (513 CpGs)^p^, cell-count enriched Hannum clock^c^	b, s, l, m, mn	[21]: *n* = 82 subjects with high versus *n* = 131 with low hardship [22]: *n* = 100 women of African ancestry; 17.8% less than a 12th grade education [23]: *n* = 1594 subjects with low versus *n* = 1744 with high socioeconomic status (participants of three large prospective cohorts) [24]: *n* = 101 African American children (6–13 years old); 56,6% with low, versus 9,1% with high parents’ income; 57,7% with low 5% with high education level [25]: *n* = 932 persons (aged 16 years or more) [26]: life-course social economic status trajectory: *n* = 96 low/low, *n* = 85 low/high, *n* = 67 high/low, *n* = 87 high/high socioeconomic status [27]: multi-cohort study, 16,245 individuals [28]: *n* = 234 subjects above versus *n* = 236 below poverty [29]: 539 women with low versus 240 women with high education [30]: *n* = 287 children with versus *n* = 669 without financial hardship; *n* = 144 with versus *n* = 829 without neighbourhood disadvantages; 3.5–7 years old [31]: 27 cohort studies with a total of 10,767 individuals [32]: education: *n* = 4816, socioeconomic status: *n* = 4728 [33]: 458 women with low versus 1282 women with high education	**↑** (up to 1.85 years)
Experience of violence during childhood	[24, 30, 34, 35]	[24, 34] Horvath (353 CpGs)^c^, [30, 35]: Hannum (71 CpGs)^c^	s, b	[24]: *n* = 93 children with violence exposure (6–13 years old) [30]: *n* = 123 abused versus *n* = 850 unabused children (3.5–7 years old) [34]: *n* = 247 children/adolescents (8–16 years old) with wide variability in early lifetime adversity exposure [35]: multi-cohort study; *n* = 616, 20 and 22 years old	**↑**
Cancer (several types)	[2, 12, 36–41]	[2, 36–41]: Horvath (353 CpGs)^c^, [36–38, 41]: Hannum (71 CpGs)^c^, [41]: Weidner clock (3 CpGs)^b^ [12, 41]: ELOVL2^b^ [12, 41]: FHL2^b^, [12]: MIR29B2C/C1orf132^c^, TRIM59^c^, KFL14^c^	b/l	[2]: multi-data-set study of different tissues (healthy and diverse diseases, i.e. cancer) [12]: *n* = 39 patients with chronic lymphatic leukaemia versus 92 healthy controls [36]: *n* = 835 with colorectal cancer, *n* = 170 with gastric cancer, *n* = 143 with kidney cancer, *n* = 332 with lung cancer, *n* = 869 with prostate cancer, *n* = 439 with B cell lymphomas, *n* = 428 with urothelial cell carcinoma (aged between 27 and 76 years) [37]: *n* = 1864 older adults aged 50–75 years [38]: *n* = 686 (longitudinal study); *n* = 442 cancer free at first blood draw [39]: *n* = 451 female breast cancer patients versus 451 healthy controls [40]: *n* = 2029 women between the ages of 50–79 years [41]: *n* = 421 patients with diverse cancers versus 424 controls	**↑** (up to 2.5 years)
Obesity/high BMI/blood cholesterol levels	[27, 29, 32, 33, 42–44]	[27, 33]: Hannum (71 CpGs) ^c^ [27, 29]: Levine (513 CpGs) ^p^ [27, 32, 33, 42–44]: Horvath (353 CpGs) ^c^	b, bc, s	[27]: multi-cohort study, 16,245 individuals [29]: *n* = 1206 women [32]: *n* = 5200 [33] *n* = 940 women with low versus *n* = 1093 with high BMI [42]: *n* = 183; longitudinal study [43]: *n* = 790 women [44]: *n* = 72 women with normal (< 24.9), *n* = 58 overweight (BMI 25–29.9) and *n* = 102 obese (BMI ≥ 30) women	**↑** (up to 1.08 year / 0.14 years per 1 kg/m^2^ increase in BMI)
Infectious diseases	[45, 46]	Horvath (353 CpGs) ^c^	b	[45]: 3 datasets of HIV-infected (*n* = 156) versus 5 datasets from uninfected healthy subjects [46]: 137 HIV-infected vs. 44 healthy males	HIV**: ↑** (4.9/5.2 years)
	[47]		b	*n* = 122 nonagenerians (cytomegalovirus (*CMV*) seropositivity rate 95%); *n* = 21 young controls (*CMV* seropositivity rate 57%)	CMV: **↑** (up to 6 years)
	[48]	Horvath (353 CpGs) ^c^, Hannum (71 CpGs) ^c^	b	*n* = 765 *Helicobacter pylori* infected versus *n* = 712 uninfected	**↑** (up to 1 year)
Alcohol consumption	[27, 33, 49, 50]	[27, 33, 49]: Horvath (353 CpGs) ^c^, [27, 33]: Hannum (71 CpGs)^c^, [27, 50]: Levine (513 CpGs) ^p^	b	[27]: multi-cohort study, *n* = 16,245 individuals [33]: *n* = 1944 subjects drinking less than 1 versus *n* = 579 subjects drinking more than 3 drinks/week [49]: *n* = 202 individuals with alcohol dependence versus *n* = 256 healthy controls [50]: *n* = 331 individuals with alcohol use disorder versus *n* = 201 healthy controls	**↓** with moderate alcohol consumption **↑** (up to 2.2 years) with habitual alcohol consumption / dependence / alcohol use disorder
Smoking	[27, 33, 40, 51–53]	[27, 33, 51]: Horvath (353 CpGs) ^c^, [27, 33, 51]: Hannum (71 CpGs) ^c^, [27, 53] Levine (513 CpGs) ^p^, [52]: 8 CpG-clock ^b^	b, bc	[27]: multi-cohort study, *n* = 16,245 individuals [33]: *n* = 479 smokers versus *n* = 4394 non-smokers [40]: *n* = 925 smokers versus *n* = 1104 non-smokers (50–79 years old) [51]: *n* = 279 current and *n* = 510 former versus *n* = 720 never smokes (50–75 years old) [52]: *n* = 200 current, *n* = 229 former and *n* = 67 occasional versus *n* = 193 never smokers [53]: *n* = 692 males; 66% former, 30% never, 4% current smokers	Inconsistent; *trend* **↑**
Exposure to environmental toxins	[54–59]	[54–57, 59]: Horvath (353 CpGs) ^c^, [57–59]: Hannum (71 CpGs) ^c^, [55]: Levine (513 CpGs) ^p^, 239 CpG-clock ^c^	b, bu	[54]: longitudinal study; baseline *n* = 4.261, follow-up *n* = 1777 [55]: 3 panels of Chinese populations, *n* = 539 [56]: longitudinal study; *n* = 589 males [57]: longitudinal study; *n* = 552 [58]: *n* = 1000 [59]: longitudinal study; *n* = 2747 women	Mainly **↑** (up to 6 years)
Sex	[3, 28, 32, 60, 61]	[3, 28, 61]: Hannum (71 CpGs) ^c^, [28, 32, 60, 61]: Horvath (353 CpGs) ^c^	b, s	[3]: *n* = 656 subjects, 19–101 years old [28]: *n* = 238 males versus 232 females (middle-aged) [32]: *n* = 1918 males versus 3083 females [60]: *n* = 1523 males versus *n* = 2775 females from 12 different cohorts [61]: *n* = 495 boys versus *n* = 527 girls; longitudinal study	**↑** in males (~ 1 year)
Ethnicity	[9, 28, 29, 60, 62]	[9]: 5 CpGs (ELOVL2, C1orf132, TRIM59, KLF14, FHL2) ^c^ [28, 60, 62]: Horvath (353 CpGs) ^c^, [28]: Hannum (71 CpGs) ^c^ [29]: Levine (513 CpGs)^p^	b,s	[9]: *n* = 100 Koreans (20–74 years) versus *n* = 300 subjects from Poland (from [63]) [28]: *n* = 244 African American versus *n* = 243 white middle aged subjects [29]: *n* = 310 black and *n* = 222 Hispanic versus *n* = 662 white women [60]: *n* = 708 subjects of European versus *n* = 2711 subjects of Indian ancestry [62]: *n* = 204 African American versus *n* = 161 European American newborns	*EA differs between ethnicities* (e.g. Hispanics and Tsimane Amerindians > Caucasians, African Americans < Caucasians and Hispanics)

**Table 2:** Other factors with effects on epigenetic age. *K* kind of sample: *b* blood, *mn* mononuclear cells

Factor	Reference	Age prediction model (referring to chronological (c), phenotypic (p) age)	K	Information on investigated populations (as far as available)	Influence on the estimation of chronological age
Insomnia	[64]	Horvath (353 CpGs)^c^, enhanced Hannum model (71 CpGs + blood cell counts)^c^	b	*n* = 411 women with versus *n* = 1667 without insomnia symptoms	**↑** (up to 1.77 years)
Night shift work	[65]	Horvath (353 CpGs)^c^, Hannum (71 CpGs)^c^, Levine (513 CpGs)^p^	b	*n* = 175 (have been) working nightshift, *n* = 2399 never worked nights shift (women aged 35–74 years)	**↑** (~ 3 years)
Parental depression at child age 11	[66]	Horvath (353 CpGs)^c^	b mn	*n* = 242 11-year-old children with versus *n* = 157 without intervention	**↑** (at child age 20)
Early onset Alzheimer’s disease	[11]	5 CpGs (ELOVL2, C1orf132, KLF14, FHL2, TRIM59)^c^	b	*n* = 31 early onset Alzheimer’s disease patients (aged 31–44 years) versus *n* = 120 healthy controls	**↑** (mean 6.5 years)
Graves’ disease				*n* = 44 Graves’ disease patients (aged 12–30 years) versus *n* = 120 healthy controls	**↑** (mean 3.7 years)
Parkinson’s disease	[67]	5 CpGs (ELOVL2, C1orf132, KLF14, FHL2, TRIM59)^c^ Horvath (353 CpGs)^c^	b	*n* = 335 with versus *n* = 257 without Parkinson’s disease (35–92 years old)	**↑**
Hutchinson Gilford progeria syndrome	[68]	Horvath skin and blood clock (391 CpGs)^c^	b	*n* = 14^*^ Hutchinson Gilford progeria patients	**↑**
Sotos, Rett and Kabuki syndrome	[69]	Horvath (353 CpGs) ^c^	b	Sotos syndrome: *n* = 20* Rett syndrome: *n* = 15* Kabuki syndrome: *n* = 46* Healthy: *n* = 1128	Sotos: **↑** (7.6 years) Rett: **↑** (2.7 years) Kabuki: **↓** (1.8 years)
Nutrition	[33]	Horvath (353 CpGs)^c^ Horvath (353 CpGs)^c^, Hannum (71 CpGs)^c^	b	*n* = 704 women with low versus *n* = 1156 with high fish consumption; *n* = 518 women with high versus *n* = 579 with low blood carotenoid levels; *n* = 748 women with high vs *n* = 826 with low poultry consumption	**↓** *healthy* (high carotenoids, fish) (up to 3.3 years) **↑** *high poultry consumption*
Longevity of parents	[70]	Horvath (353 CpGs)^c^	b mn	*n* = 63* semi-supercentenarians’ offspring versus *n* = 47 controls	**↓** offspring (5.1 years)
Intense physical exercise	[10]	5 CpG clock (ELOVL2, C1orf132, KLF14, FHL2, TRIM59) ^c^, 2CpG-clock (TRIM599, KLF14) ^c^	b	*n* = 176 athletes versus *n* = 128 age- and gender-matched controls	**↑** (up to 5.9 years)
Abnormal glucose tolerance during pregnancy	[71]	Hannum (71 CpGs) ^c^, Levine (513 CpGs) ^p^	b	*n* = 113 women with abnormal versus *n* = 2243 women with normal glucose tolerance during pregnancy	↑ (mother**)**

*n lower than 90 due to rarity of factor

## Reported factors with effects on epigenetic age

Parameters that were repeatedly shown to impact epigenetic age-predictions were particularly observed in the following categories: Lifetime adversities (chronic stress, trauma/PTSD, exposure to violence, low socioeconomic status/education), cancer, obesity and related diseases, insomnia, exposure to toxins (alcohol, smoking, air pollution, pesticides), sex and ethnicity (Table 1). Other factors so far have only been described once (Table 2).

### Impact of lifetime adversities on epigenetic age

The effects of adverse living conditions have been addressed by numerous publications. Cumulative *lifetime stress* results in an increased epigenetic age measured via Horvath clock [14]. This effect was especially pronounced in advanced age. Moreover, severe stress-related diseases (*psychotraumata* or post-traumatic stress disorder (*PTSD*)) may lead to increased epigenetic age estimation. Boks et al. [15] found increased epigenetic age estimates in 96 traumatized male soldiers based on the application of the Horvath clock. Wolf and colleagues [16–18] investigated the effects of childhood or lifetime trauma: Regarding the Horvath clock, they did not find effects of PTSD and childhood trauma on epigenetic age. This finding was supported by Mehta et al. [19]. In contrast, a correlation between increased epigenetic age estimation and childhood trauma as well as lifetime PTSD severity was evident with the Hannum clock. The data presented in a meta-analysis [20] hint to a higher impact of traumata experienced during childhood compared with those experienced in adulthood.

A low *socioeconomic status* and/or *education* level may also be associated with increased epigenetic age estimations. Chen et al. [21] investigated epigenetic ages of 379 African American adolescents in Georgia by analysing DNA from leukocytes. The epigenetic age of those individuals was increased by 1.42 years per measure of economic adversity. Another group [22] reported increased epigenetic age estimates in black middle-aged women with low income. They also investigated “the extent to which various health-related behaviours such as diet, exercise, smoking, alcohol consumption, and having health insurance could explain the effect of income on aging”, but did not find “significant relationship between these variables and the speed to which aging occurred, and controlling for them had no impact on the association between income and biological aging.” Fiorito et al. [23] described increased epigenetic age estimation in individuals with low socioeconomic status. The authors pointed out that this effect could be ameliorated by increasing socioeconomic status during life course; however, epigenetic age estimates still remained higher in those individuals compared with those with higher socioeconomic status. Jovanovic et al. [24] investigated DNA isolated from saliva of children and found an association between increased epigenetic age estimates and low socioeconomic status. These results are supported by the findings of Hughes et al. [25], who investigated the epigenetic age of individuals whose parents had been in semiskilled or unskilled occupations when the investigated individuals were 14 years old. At an age of 26 years, these individuals exhibited an increased epigenetic age of 1.07 years. In individuals without a working parent at an age of 14 years, this effect was even more severe, resulting in an increase of epigenetic age estimates of 1.85 years at an age of 26 years. The authors state that these “differences were not explained by smoking, adiposity or alcohol consumption, suggesting mechanisms independent of health behaviours are involved.” These results are again supported by the data of Austin et al. [26], who reported an increase in epigenetic age in individuals with low early life socioeconomic status, as well as by Fiorito et al. [27], Tajuddin et al. [28], Thurston et al. [29] (regarding education) and Marini et al. [30] (regarding children), each of them with application of varying epigenetic clocks. Fiorito et al. [27] stated again that the increases of epigenetic age with low socioeconomic status were mainly independent of other lifestyle-related risk factors like smoking, obesity, alcohol intake, and low levels of physical activity. It is not clear yet, why a low socioeconomic status alters epigenetic age. Some groups assumed that this might be due to the stress of experiencing economic hardship, and that changes of DNA methylation are mediators of the association of low socioeconomic situation and a higher risk of diseases (e.g. cardiovascular diseases) [24, 26, 72, 73].

In contrast, high socioeconomic position or education levels have been shown to be associated with reduced epigenetic age estimates [31]. McCartney et al. [32] reported that each degree of increase in education level or socioeconomic status led to 0.05 years decrease of epigenetic age estimates. Additionally, Quach et al. [33] stated that higher education is associated with a decrease in epigenetic age estimates (4.14 years) and also described an inverse relationship between income and epigenetic age.

*Exposure to violence* was also suggested to impact epigenetic age. Based on the analysis of DNA isolated from saliva of children, Jovanovic et al. [24] reported an association between an increase of epigenetic age estimates and experiences of violence in a dose dependent manner. Similarly, Sumner et al. [34] found increased epigenetic age estimates and an advanced pubertal stage in children with threat-related early life adversity (e.g. violence). These results were supported by Marini at al. [30], who found “that exposure to abuse, financial hardship, or neighbourhood disadvantage during sensitive periods in early and middle childhood (…) (led to a) deviation of Hannum based epigenetic age from chronological age, even after considering the role of adversity accumulation and recency”. Regarding Horvath clock, they did not find differences between estimated ages of children with or without exposure to violence. Brody et al. [35] described the effects of exposure to higher levels of racial discrimination on epigenetic age estimates and reported increased epigenetic age estimates in discriminated adolescents with less supportive families (known to ameliorate the effects of exposure to racial discrimination).

In forensic case work, age estimation in young migrants without valid identity documents has become highly relevant in these times of migration and flight [74]. In this context, the possible impact of adverse living conditions on epigenetic age estimation is of major importance. If an accumulation of stressors like experiences of violence and low socioeconomic status may cause increased epigenetic age estimates and, consequently, a false high age estimate, this may have serious consequences for the individual (e.g. legal responsibility).

### Epigenetic clocks may be distorted in non-infectious diseases

Spolnicka et al. [11] tested an epigenetic model composed of five markers from five genes (*ELOVL2*, *C1orf132*, *KLF14*, *FHL2* and *TRIM59*) for the estimation of chronological age in three disease groups (late and early onset Alzheimer’s disease, Graves’ disease). They reported aberrant hypermethylation and decreased prediction accuracy of chronological age for *TRIM59* and *KLF14* markers in the group of early onset Alzheimer’s disease. In Graves’ disease patients, an aberrant hypermethylation was observed for *TRIM59*, an aberrant hypomethylation for *FHL2*. In contrast, *ELOVL2* and *C1orf132* showed unchanged prediction accuracy in all disease groups. Analysis of the identical five markers in 39 blood samples from patients with chronic lymphocytic leukaemia (CLL) and 92 healthy individuals (control group) resulted in highly statistically differences between patients and controls for all CpGs, indicating a strong influence of CLL on age-related methylation [12]. The authors concluded that “DNA methylation signature in blood does not predict calendar age in patients with chronic lymphocytic leukemia”.

Altered DNA methylation patterns were not only observed in cancer which affects blood cell lines [12], but also in other common types of cancer like colorectal, gastric, kidney, lung, prostate and urothelial cancer [2, 36–41]. Dugue et al. [36] found that “epigenetic aging was associated with increased cancer risk, ranging from 4% to 9% per five-year age acceleration”. Several other publications reported associations between cancer/cancer risk and an increase of epigenetic age estimates in blood of up to 2.5 years [37–41]. In fact, cancer is a monoclonal disease and hence the malignant cells capture the epigenetic makeup of the tumour initiating cell [75]. There is even evidence that for many types of cancer, the acceleration of epigenetic age is of prognostic value for disease development [76]. However, it is unknown so far if increased epigenetic age is a cause or consequence of cancer development. The aforementioned data are also of relevance for forensic casework: Blood samples of persons suffering from cancer may exhibit altered DNA-methylation levels that might result in false high age estimates. This may also be true if the cancer already exists, but is not diagnosed yet.

### Infectious diseases impact epigenetic age predictions

Based on the DNA-methylation pattern in blood, epigenetic age of individuals infected with HIV was estimated 5.2 years higher compared with healthy controls [45]. These results were confirmed by Gross and colleagues [46]. Similarly, *H. pylori* and *Cytomegalovirus* (CMV) infections were associated with increased epigenetic age estimates [47, 48]. In forensic casework, the impact of those infections on epigenetic age estimates has to be kept in mind, as they might result in deviations between estimated and chronological ages of up to 6 years. This point is of high importance regarding age estimation of unaccompanied young refugees without valid documents, as many refugees stem from African regions with high HIV-incidence, and additionally CMV infections are very common with up to 95% incidence in adults in many countries [77].

### Obesity fosters epigenetic ageing

Obesity, high body mass index (BMI), and blood cholesterol have been shown to increase epigenetic age estimates. Nevalainen et al. [42] found increased epigenetic age estimates in middle-aged individuals with high BMI. These findings were supported by data of Simpkin et al. [43], McCartney et al. [32], Li et al. [44] and Thurston et al. [29]. Recently, Fiorito et al. [27] published data from a large multi-cohort study (*n* = 16,245), revealing increased epigenetic age estimates of up to 1.08 years (*p* < 0.001) for individuals with high BMI (BMI ≥ 30). Individuals with lower BMI may exhibit low epigenetic ages [33].

### Alcohol, smoking, and environmental toxins affect epigenetic ageing

Moderate *alcohol* consumption (1–7 drinks/week) was suggested to decrease epigenetic age [33]. In contrast, habitual alcohol consumption [27] and alcohol dependence [49] were associated with an increase in epigenetic age. These data were recently supported by Luo et al. [50].

There is clear evidence that *smoking* evokes specific DNA methylation changes [78]. The data available for the impact of smoking on epigenetic age estimations are still inconsistent. Some groups did not report any effects [33, 40, 51, 52]. Studies comprising larger sample sizes report increased epigenetic age estimates in smokers: McCartney et al. [32] investigated DNA from 5100 blood samples and found increased epigenetic age estimates in smokers. Fiorito et al. [27] investigated 16,245 blood samples and reported that smoking was associated with an increased epigenetic estimate of up to 1.57 years. Additionally, Yang et al. [53] reported that cumulative smoking (pack-years) was significantly associated with epigenetic acceleration.

Even exposure to *environmental toxins* has been shown to affect epigenetic age. Ward-Caviness et al. [54] reported an increase of epigenetic age with increased exposure to fine particulate matter and nitrogen oxide. Similarly, Li et al. [55] as well as Nwanaji-Enwerem et al. [56, 57] found increased epigenetic ages after exposure to black carbon or fine particulate matter. Lind et al. [58] described increased epigenetic age estimates in individuals exposed to pesticides. In contrast to this, White et al. [59] found a deceleration of the epigenetic age after exposure to NO_2_. However, the age acceleration effect of fine particulate matter varied from 2 to 6 years and depends on its composition.

### Epigenetic ageing is faster in men compared with women

In age estimation models comprising of high numbers of markers, males are estimated older than females of identical age. This finding was first described by Hannum et al. [3], who found that—on the epigenetic level—men age approximately 4% faster than women. These results were supported by the findings of other studies based on different models for age estimation [28, 32, 60, 61]. Notably, no difference in epigenetic age estimates between men and women were reported in one study based on a model comprising of only 8 CpGs [52].

### There may be differences in epigenetic ageing between ethnic groups

Cho et al. [9] applied an epigenetic model for the estimation of chronological age (markers located in the *ELOVL2*, *C1orf132*, *TRIM59*, *KLF14*, and *FHL2* genes) derived from a Polish population [63] to blood samples from 100 Koreans. They reported that the age predictive performance of the tested model was “relatively consistent across different population groups”. However, at certain loci (*FHL2*, *C1orf132*, *KLF14*) the extent of the age association in Koreans was not identical to that of the Polish, and retraining of the models produced better prediction accuracy. Several other publications describe differences in epigenetic age estimates between same aged individuals of different ethnic groups, too [28, 29, 60, 62]. As an example, Hispanics and Tsimane Amerindians have been shown to exhibit accelerated epigenetic ageing compared with Caucasians, while African-Americans were suggested to have decelerated epigenetic ageing compared with Caucasians and Hispanics [60].

## Other factors with effects on epigenetic age

Spolnicka [10] investigated the effects of intense *physical exercise* on DNA methylation and its impact on epigenetic estimation of chronological age: elite athletes exhibited accelerated DNA hypermethylation of *TRIM59* and *KLF14*; both markers predicted the athletes to be several years older than controls (*KLF14*: on average 5.5 years older, *TRIM59*: on average 4.5 years older) [10].

*Insomnia* [64] and working *night shift* [65] were reported to increase epigenetic age estimates. Children experiencing a *parent’s depression* at an age of 11 years exhibited increased epigenetic ages at an age of 20 years [66]. This effect could be ameliorated by psychosocial intervention for those children. Increased epigenetic age estimates have also been reported in patients suffering from age related diseases like *Parkinson’s disease* [67]. Rare diseases like *Hutchinson Gilford progeria*, *Sotos*, *Rett* and *Kabuki syndromes* were also shown to affect epigenetic ages (up to + 7.6 years (Sotos syndrome)) [68, 69].

Other factors have been shown to decrease epigenetic age estimates, such as healthy *nutrition* (vegetables, fruit, fish) [33]. Additionally, Horvath et al. [70] reported an interesting association between epigenetic age estimates in individuals and *longevity of* their *parents*: children of 105–110-year-old parents were estimated significantly younger (however, this was only investigated in a small number of Italian individuals). The effects of *physical activity* on epigenetic age estimates seem to depend on the intensity of sports: elite athletes who exercised very intensely showed increased epigenetic age estimates [10], whereas leisure time physical activity did not affect epigenetic age estimates [79]. In contrast, samples from individuals with low physical activity (low score on International Physical Activity Questionnaire, LASA Physical Activity questionnaire, low Cambridge Physical Activity Index, sedentary job and no recreational activity or < 1 h/week of physical activity) showed a trend towards a slight increase in epigenetic age estimation [27]. Additionally, abnormal glucose tolerance during pregnancy has been shown to lead to increased epigenetic age estimates of mothers [71].

## Conclusions

Epigenetics of ageing is an emerging field for forensic application, opening perspectives to estimate the chronological age of living persons, deceased persons, or of blood traces. The choice of relevant age-associated CpGs and of the method applied for DNA methylation analysis need to be adjusted to the specific needs. Targeted assays, e.g. by pyrosequencing, digital droplet PCR, or bisulfite barcoded sequencing, may provide cost-effective and robust alternatives to the frequently used Illumina BeadChip arrays [13, 80]. The development of prediction models should consider the reality of casework (e.g. high-quality and high-quantity DNA samples versus low-quality and low-quantity DNA, e.g. from blood traces). Additionally, the method-to method-bias has to be addressed.

It becomes more and more evident that epigenetic ageing is influenced by diverse exogenous and endogenous factors. Knowledge of these factors and of their impact is highly relevant for epigenetic age estimation—in forensic casework as well as in research. This review summarizes factors that may be relevant in forensic context. Although not all of them have been conclusively proven yet, they should at least be regarded as potential confounding factors that may contribute to the sometimes high deviations between chronological age and epigenetic age predictions. In fact, the impact of such exogenous and endogenous parameters needs to be explored regarding each specific forensic application, for each choice of relevant age-associated CpGs that are considered for age-predictions, and for each method that is utilized for DNA methylation measurement.

Future research has to address also the risk of confounding biological factors and should focus on (1) the identification of forensically relevant biological/environmental confounding factors and (2) on the development of models that are “robust” against the influence of such biological factors.

### Identification of forensically relevant biological confounding factors

Facing a growing list of factors influencing DNA methylation, the identification of relevant factors in the forensic context (estimation of chronological age) is of great importance. A factor is relevant, if it may cause significant and systematic deviations of epigenetic age estimates from chronological age and if it is likely to be present in forensic cases.

A first step towards the identification of relevant factors can be a pre-selection by analysis of published literature including clinical and basic science data, as done in this review. Taking into account the data in Tables 1 and 2, such pre-selected factors could be—for example—*CMV* infections (differences between epigenetic and chronological ages of ~ 5 years, high incidences especially of *CMV* infections).

In a second step, the influence of these factors on the quality of age estimation by models used for forensic age predictions can be tested by a targeted investigation of populations bearing possible confounding factors—a strategy that has already been chosen by Spolnicka et al. to test the relevance of physical activity and selected diseases [10–12].

However, some confounding factors may be interrelated (e.g. BMI, nutrition, low fitness and low socioeconomic status) and the individual impact of each of these interrelated factors can possibly not be differentiated clearly, since an independent investigation of them appears difficult.

The identification of forensically relevant factors, however, does not solve the question of how one can recognize if such a factor has to be taken into account in the individual forensic case. This problem arises especially in postmortem cases (unidentified deceased) and the analysis of blood traces of unknown donors for crime scene investigations. Spolnicka et al. [12] proposed a classification model for the identification of patients with chronic lymphocytic leukaemia (which has a strong impact on DNA methylation), deduced from the DNA methylation data. In living individuals, age estimation should consider the detailed medical history; specific questions should be asked about possible factors of influence.

### Development of models that are “robust” against the influence of confounding biological factors

Models that are as robust against the influence of confounding biological factors as possible have to be developed to minimize the influences of the identified confounding factors on the quality of forensic age estimation.

Theoretically, models that include large numbers of CpGs could have the advantage that the influence of confounding biological factors on some markers may be “compensated” by an unaffected DNA methylation of other CpGs. However, even “large clocks” may be influenced by confounding biological factors (see “Horvath clock” (353 CpGs) and “Hannum clock” (71 CpGs) in Tables 1 and 2). The same is true for epigenetic models composed of a small number of CpGs, as shown for example by Spolnicka et al. [10] (5 CpGs). Within such models, some DNA methylation markers may be more sensitive towards confounding biological factors than others may, as shown for example for *ELOVL2* and *C1orf132* in three disease groups [11]. Focusing on a selected number of such “robust” CpGs in targeted assays may be a promising approach for the development of “robust” models for epigenetic age estimation; such “specialized” clocks [13, 80] may be much more powerful for forensic age estimation. This approach will result in models composed on only few CpGs, which will also be applicable in settings with low amounts of DNA of good quality. Another approach may be the consideration of identified confounding factors as parameters in prediction models.

The aim of optimization of epigenetic age estimation poses many challenges. Apart from aspects like the quality of DNA, applicability and suitability of methods, predictive markers, and integration of DNA methylation measurements into predictive models [80], it is important to elucidate how biological confounding factors affect epigenetic age-predictions. This interplay needs to be understood for reliable applications in forensic contexts. Collaborative research with coordinated research strategies is required to address this multitude of open research questions and to improve epigenetic methods for the estimation of chronological age.
